# Military traumatic brain injury: a challenge straddling neurology and psychiatry

**DOI:** 10.1186/s40779-021-00363-y

**Published:** 2022-01-06

**Authors:** Ling-Zhuo Kong, Rui-Li Zhang, Shao-Hua Hu, Jian-Bo Lai

**Affiliations:** 1 Department of Psychiatry, The First Affiliated Hospital, Zhejiang University School of Medicine, Hangzhou, 310003 China; 2 The Key Laboratory of Mental Disorder’s Management in Zhejiang Province, Hangzhou, 310003 China; 3 Brain Research Institute of Zhejiang University, Hangzhou, 310003 China; 4 Zhejiang Engineering Center for Mathematical Mental Health, Hangzhou, 310003 China; 5 MOE Frontier Science Center for Brain Science and Brain-Machine Integration, Zhejiang University, Hangzhou, 310003 China

**Keywords:** Shellshock, Military, Traumatic brain injury, Diagnosis, Treatment, Comorbidity

## Abstract

Military psychiatry, a new subcategory of psychiatry, has become an invaluable, intangible effect of the war. In this review, we begin by examining related military research, summarizing the related epidemiological data, neuropathology, and the research achievements of diagnosis and treatment technology, and discussing its comorbidity and sequelae. To date, advances in neuroimaging and molecular biology have greatly boosted the studies on military traumatic brain injury (TBI). In particular, in terms of pathophysiological mechanisms, several preclinical studies have identified abnormal protein accumulation, blood–brain barrier damage, and brain metabolism abnormalities involved in the development of TBI. As an important concept in the field of psychiatry, TBI is based on organic injury, which is largely different from many other mental disorders. Therefore, military TBI is both neuropathic and psychopathic, and is an emerging challenge at the intersection of neurology and psychiatry.

## Background

Head injuries caused by bullet penetration, violent impact, or shock waves from explosive weapons are the main causes of military traumatic brain injury (TBI), which had long been under debate for being an organic disease (a neurological disease) or a functional disease (a psychiatric disorder).

During World War I, “shellshock” first came to public attention as a representative military TBI. In 1915, Myers [1] published a paper in *The Lancet* that established the medical status of shellshock, which was described as "concussion" in the following year by Mott [2], who then proposed the pathological hypothesis in 1917. However, due to various ethical issues, the diagnosis of shellshock was later restricted. In 1939, the diagnosis of shellshock was officially abolished. In 1943, psychiatry began to receive attention in the military field, and carried out a study on casualties in the 34th and 45th Divisions of the U.S. and the Western Pacific and Mediterranean theatres, which proved the correlation between the number of wounds and the number of psychiatric hospitalizations [3]. It was not until 1980, when the concept of posttraumatic stress disorder was established, that military TBI was officially made public again. Twelve years later, the term "mild traumatic brain injury (mTBI)" firstly came into being. mTBI—an updated and expanded version of the old concept of "shellshock"—was officially accepted by the public as a definitive diagnosis of traumatic mental disorder in 2005, when it was used to describe the effects of explosive weapons on American soldiers serving in Iraq and Afghanistan.

mTBI is based on neurotrauma, which is located at the intersection of neurology and psychiatry. Currently, while mTBI is conceptually similar to concussion, moderate and severe TBI mainly refers to penetrating brain injury. In addition, the concept of chronic traumatic encephalopathy (CTE) was first proposed in 1957 by the pathologist Harrison Matland from observations in prize boxers [4], whose main clinical symptoms and signs were dementia-like manifestations, neuromuscular dysfunction, and psychosis [5]. However, not until 2005 did academic circles widely recognize the concept of CTE [6].

## Military TBI: epidemiology and mechanism

### Shocking figures

Despite the rapid advancements in weaponry, innovations in the methods of combat, and the growing trend towards unmanned warfare, it is undeniable that a reduction in the number of wounded soldiers does not equal a decrease in the percentage of soldiers suffering from TBI. In reality, the prevalence of TBI in modern military operations is still staggering. According to the U.S. Centres for Disease Control and Prevention, the number of TBI cases in the U.S. increased dramatically between 2000 and 2006, due to the return of large numbers of soldiers stationed overseas [7]. The U.S. Armed Forces Health Monitoring Centre reported that 375,230 U.S. servicemen suffered from TBI from 2000 to 2016 [8].

Although craniocerebral injuries often give the intuitive impression of being "more severe," the majority of military TBIs that can be clinically diagnosed are mild and are quite similar to the definition of "concussion". According to previous studies, generally speaking, the incidence of mTBI among soldiers who participated in Operation Iraqi Freedom (OIF), Operation Enduring Freedom (OEF) and Operation New Dawn (OND) deployment reached 15.2–22.8% [9], and the most important cause of brain injury was the long-term exposure to explosive weapons [9]. Of the 244,217 cases of TBI reported by the Defence Medical Surveillance System and Theatre Data Storage in the U.S. from 2001 to 2012, approximately 75% were classified as mild [10] and were similar to the data released by the U.S. Defence and Veterans' Centre for Brain Injury. In 2009, Terrio et al. [11] found that 82.5% of TBI patients (*n* = 907) who had served in the military were mTBI. In the same year, the U.S. Department of Defence reported a total of 28,958 cases of military TBI. After comprehensive scoring based on the severity and causes of injury, the proportion of patients with mTBI was as high as 83% [12].

However, the prevalence of mTBI reported in the U.K., Canada and other countries participating in OIF or OEF was significantly lower than the figures presented above. According to data provided by the British Armed Forces, the prevalence of mTBI among service members during OIF and OEF was 4.4% [13], while the Canadian Armed Forces reported that 5.2% of Canadian service members deployed in Afghanistan during OEF suffered from mTBI [14]. This phenomenon may be explained by the differences in regions, modes of operation, subjects of investigation and other factors, but the exact causes remain unclear.

### Gender differences

The gender effect on TBI was mainly manifested in the difference in morbidity. Generally, the incidence of TBI in males appeared to be approximately twice as high as that in females [15], and although these data included both military and civilian TBI cases, it can be inferred that there is also a significant gender difference in patients with TBI. This is because men tend to make up the majority of military deployments, and women are less likely to be sent to the front lines of combat. A systematic review of veteran patients with TBI showed that, compared with men, women were less likely to suffer from TBI, and the incidence of emergency craniectomy was also lower. However, data regarding the incidence difference of long-term post TBI syndrome in male and female service members are still inadequate [16].

### Aetiological mechanism

On the whole, the causes of TBI are numerous, but in the military field, severe head impact, explosive device exposure, and penetrating ballistic injuries are still recognized as the major causes. On the one hand, these three types of traumas remain the most common causes of injury in modern warfare and other types of armed conflict. On the other hand, since TBI is derived from the concept of shellshock, it is hard to avoid being influenced by its original narrow definition of craniocerebral injury caused by explosive weapons. The common type of military TBI is penetrating ballistic injury, caused by instantaneous energy release with physical destruction of the neuronal fibre bundle, and explosive injury, due to intracranial vascular fluctuation, pressure gradient formation and dynamic deformation of the skull [17]. In contrast, brain injury caused by a blow to the head is less military-specific. Explosive injury is classified into four subtypes, namely, primary, secondary and tertiary injury directly or indirectly due to shock waves, with the fourth one caused by high temperature and poisonous gas released during the explosion [10]. A shock wave leads to blast wind, which then elicits an acceleration of the soldier’s body. Generally, primary injury is directly caused by the shock wave itself, while the secondary injury is due to fragments of debris propelled by the explosion. Tertiary injury, the main type of military TBI, is indirectly caused by the acceleration of the whole or part of the body due to the shock wave [18]. In fact, the vast majority of clinically diagnosed military TBI cases belong to the tertiary injury mechanism, which partly reflects the original definition of shellshock. Due to the huge power of explosive weapons, soldiers who suffered from primary and secondary explosion injuries were often killed on the spot or left with serious physical dysfunction (such as a vegetative state). Soldiers with tertiary injury are often treated as a priority for their physical wounds, such as burns of the skin and mucous membrane of the respiratory tract, or systemic toxic reactions and suffocation. Compared with visible/detectable severe trauma, the signs and symptoms of mTBI lack specificity. Therefore, it is understandable that mTBI is often omitted by clinicians. In addition, although existing bulletproof equipment can reduce the incidence of penetrating ballistic injuries to a certain extent, it also amplifies the shockwave effect, thus becoming one of the major causes of TBI in explosions [18].

**Fig. 1:**
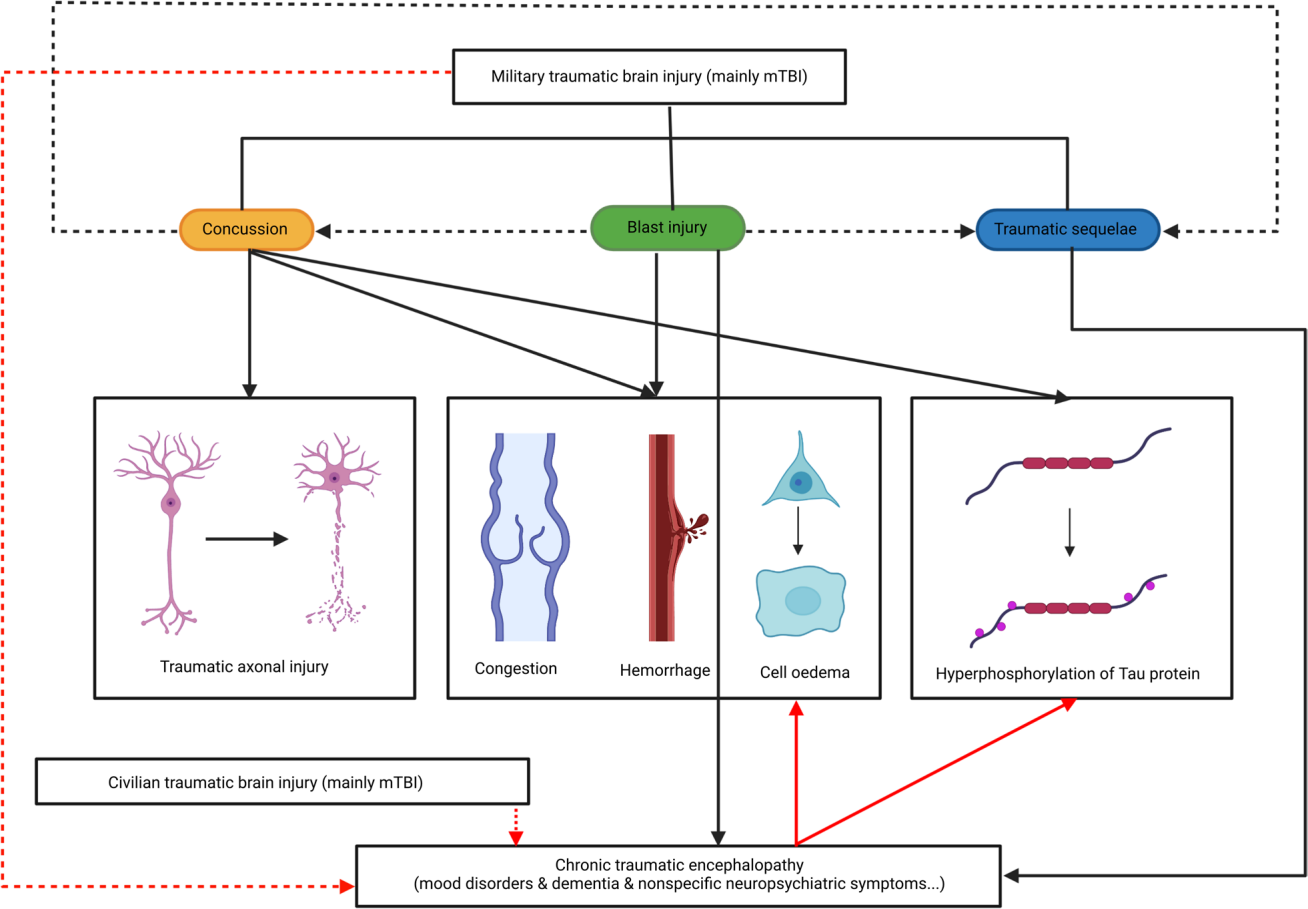
Relationship between concussion, blast injury, post-traumatic brain sequelae and chronic traumatic encephalopathy (CTE). The broad definition of military TBI can be divided into concussion, blast injury and traumatic sequelae. Among them, concussion can directly or indirectly cause damage of neuronal axon, congestion, haemorrhage, cell oedema and hyperphosphorylation of Tau protein. The blast injury mainly resulted in congestion, haemorrhage and cell oedema. Blast injuries can be the cause of concussion and traumatic sequelae. Concussions can also cause traumatic sequelae. The main cause of CTE is traumatic sequelae. Blast injury can also lead to CTE to a certain extent, while concussion has little relationship with CTE. The pathological manifestations of CTE are mainly congestion, haemorrhage, cell oedema, and hyperphosphorylation of Tau protein. Macroscopically, both military and civilian TBI can be the cause of CTE. Solid black and red arrows indicate associations of pathological mechanisms or clinical manifestations, dashed black arrows indicate relationships among subtypes, and dashed red arrows indicate possible aetiology of CTE. mTBI mild traumatic brain injury

According to the severity and characteristics of the pathological changes, the narrow sense of military TBI refers to concussion, blast injury and functional sequelae of the traumatic brain. Of the three, mTBI is the leading cause of those injuries. It should be noted that concussion is a common type of mTBI. It is relatively narrow in scope because the concept is defined on a more nuanced and specific level (pathology). In particular, CTE shows the characteristics of a progressive disease course [19]. Although the neuropsychiatric symptoms of functional sequelae of the traumatic brain can last for a long period, its clinical manifestations of progression can be inapparent, which is why we need to distinguish functional sequelae of the traumatic brain from CTE. In fact, although not all concussions lead to CTE and many victims recover over time, most cases with persistent CTE have a history of multiple concussions. At the same time, although a considerable amount of epidemiological research has been obtained, findings on the pathological mechanism and prognosis of CTE are mostly based on the general population. However, there are only a few studies in the military field, mainly because the disease is more commonly noticed in boxing and football or other professional sports that can easily lead to trauma of the head. Therefore, we consider CTE a type of trauma independent of the narrow concept of military TBI, although its aetiology, pathogenesis, clinical manifestations and pathological features have much in common with military TBI (Fig. 1). Additionally, in view of the extensive comorbidities between functional sequelae of the traumatic brain and other types of neuropsychiatric diseases such as epilepsy, dementia, PTSD and depression, it is difficult to distinguish the sequelae of brain injury from other brain functional disorders by the clinical symptoms alone, and this will be explained in detail below.

#### Concussion and blast injury

A concussion is defined as a temporary impairment of brain function caused by trauma to the head; the duration of the accompanying memory loss is mostly less than 24 h [20], and the vast majority of patients will achieve complete relief of symptoms within 7–10 days [21]. A commonly used psychometric tool for assessing the severity of concussion is the Glasgow Coma Scale (GCS), which mainly evaluates verbal and motor responses, as well as the ability to open eyes, and it can be used to initially determine the severity of concussion. The cumulative GCS score is divided into three levels of brain function damage from low to high (3–8: severe; 9–12: moderate; 13–15: mild). Although some limitations exist, the GCS is still commonly used to evaluate the consciousness of patients with TBI [22].

In the military environment, the most common mechanism of concussion is deceleration injury (i.e., blunt impact to the head) or the active impact of the head on another hard object due to a "whiplash" mechanism—for instance, a soldier's head was hit within the range of an explosive weapon [23]. Under intense force, variable motion occurs instantly in the brain tissue, including rotary motion, thus causing axon and microvascular damage [24], unbalanced ratios of ion concentrations inside and outside brain tissue cells [25], and an accelerated rate of glucose metabolism in neurons [26]. Eventually, the integrity of the blood–brain barrier (BBB), which is mainly formed by astrocytes and pericytes, is destroyed, resulting in poor perfusion of local brain functional areas [24].

In addition, it is worth noting that previous studies have shown that microvascular structure damage and astrocyte dysfunction are also common in neurodegenerative diseases such as Alzheimer's disease (AD), suggesting that concussion and AD, which is characterized by hyperphosphorylation of Tau protein, may have overlapping pathological mechanisms even though the primary cause is different [27]. Other pathological changes include microhaemorrhagic foci in brain tissue, increased astrocytes, vascular proliferation around microglia, etc. These characteristics are common signs of both concussion and AD. However, they are not specific and can also be found in other central nervous system (CNS) disorders. In particular, multifocal traumatic axonal injury (TAI) is a concept referring to the process of mechanical tearing of neurites by traumatic events (especially concussion) [28], causing mechanical damage of the axolemma and then loss of ionic homeostasis, such as disturbances in calcium homeostasis [29]. TAI finally leads to impaired axonal transport, and interruption or disconnection of axons [30].

Blast injury is the most common injury mechanism in military TBI due to the high probability of soldiers being exposed to explosive weapons. It accounts for approximately 60% of all military TBI, and as high as 80% of mTBI [31]. Shockwaves produced by explosive weapons can travel at breakneck speeds through the brain tissue [32], resulting in rapid, repetitive contraction and relaxation of brain tissue and its nutrient vessels in a very short time, causing damage to blood vessel walls and haemodynamic abnormalities, producing cerebral parenchyma and subarachnoid haemorrhage, which finally lead to pathological manifestations of brain tissue oedema [33], leading to a series of neuropsychiatric symptoms such as headache, dizziness, vomiting, and disturbance of consciousness. A recent review published in 2021 in Military Medical Research shows that another clinical symptom caused by blast injury is visual dysfunction [34].

In a study carried out in 2012, Lu et al. [35] found that after exposure to low levels of a single repeated explosion environment, brain tissues of *Macaca fascicularis* exhibited a series of pathological changes, including capillaries and blood capillary cavity collapse, hypertrophy of astrocytes, endothelial cell matrix cavitation and vascular proliferation around reticular endothelial cells. Molecular changes included an increase in aquaporin-4 (AQP4) expression in astrocytes and chromatolysis of neurons in the cerebellar cortex, hippocampal cone and Purkinje fibres. Mott [2] reported in *The Lancet* that in the case of acute blast injury, the human brain also showed pathological manifestations such as subarachnoid haemorrhage, small blood vessel congestion and bleeding in the brain parenchyma, cerebral vasospasm and mild cerebral oedema. Relatively few studies have been performed on cell metabolism related to blast injury. Peskind et al. [36] used positron emission tomography (PET) to capture images of brain glucose metabolism in veterans with repetitive explosion injuries and found a decrease in local metabolic function in the brain parenchyma. Similar results were found in a study of brain function and neuroimaging in Iraq and Afghanistan veterans [37]. McKee et al. [38] performed histopathological examination of the postmortem brain of four veterans and found that those who had been exposed to explosive weapons or devices had diffuse axonal injury, local Tau phosphorylation and nonspecific hypoxic ischaemic injury in brain tissues. In addition, these patients also reported varying neuropsychiatric symptoms, such as headache, PTSD, depression, irritability, suicidal tendency, and attention difficulties [38]. In Omalu's case, multifocal neurofibrillary tangles (NFTs) were found deep in the frontal cortex of veterans exposed to explosive weapons [39], which further demonstrated the pathologic similarity between blast injuries and concussion.

From the studies presented above, we can briefly draw the conclusion that, although blast injury is more likely to pathologically manifest as vasospasm, hyperaemia, bleeding and brain cell oedema [33] and that pathological diagnosis of concussion is focused more on multifocal TAI and hyperphosphorylation of Tau protein [27], this does not deny that these two share a homogeneous aetiology in nature. Pathological studies have confirmed that there is a conceptual crossover between concussion and blast injury; that is, blast injury can be seen as a macroscopic manifestation of concussion, and the factors that lead to blast injury can also cause a concussion.

#### Damage of BBB and cerebral oedema

In fact, there is a strong correlation between BBB dysfunction and TBI, especially mTBI. In previous studies, the mechanism of BBB damage in mTBI has been gradually clarified, including but not limited to changes in cellular signalling pathways (e.g., impairment of nitric oxide-dependent signal transduction pathways) [40], damage to microvascular structures (e.g., disruption of tight intercellular junctions and loss of pericytes) [41], and astrocyte dysfunction (e.g., caudal swelling, foot swelling, and terminal congestion) [41, 42]. Additionally, other animal studies have shown that after mTBI, functional changes of the BBB can occur in the early stage, even within 5 min after the onset of mTBI [43], but the BBB structure does not show apparent pathological changes, such as disruption of continuity or blood exudation [42]. To some extent, this finding suggests that the functional changes of the BBB after mTBI are possibly secondary, even though the time interval is short.

Increased BBB permeability is usually associated with brain oedema, whose main aetiology is oxidative damage, namely, damage to cell structure and function caused by hydroxyl radicals from hydrogen peroxide. Excessive hydrogen peroxide is derived mainly from oxygen molecules during abnormal energy metabolism [44]. Therefore, the fundamental cause of brain oedema following TBI also lies in the energy metabolism disorder of brain cells. The energy metabolism of brain cells depends on various substrates from the circulating blood, and these substances have to pass through the BBB to be absorbed and utilized by brain cells. Glucose is the main substrate of brain metabolism [45], and other metabolic substrates also include lactate, medium chain triglycerides and ketone bodies [46]. Immediately following BBB injury caused by TBI, glucose metabolism will increase temporarily due to ion imbalance, membrane potential change, and abnormal mitochondrial enzyme activity [44], and gradually decline for a long-term period [47]. This phenomenon can also be caused by a lack of glucose supply due to blocked cerebral blood flow, a reduction in brain consumption for energy, or dysfunction in the glucose transport process [44]. Preclinical studies have provided reliable evidence for these pathophysiological processes [48]. In summary, increased BBB permeability enables overloaded metabolic substrates such as glucose to reach brain cells within a short time, thus "indirectly" leading to impaired energy metabolism in brain cells—suggesting that structural changes in the BBB after TBI have a complex biphasic profile. All of these pathophysiological changes contribute to the formation of brain oedema in the development of TBI.

Typically, cerebral oedema after TBI can be divided into several stages, including cytotoxic oedema that occurs immediately after trauma and does not cause significant tissue swelling, but the subsequent stage of ionic and vasogenic oedema shows significant tissue swelling [49]. Water molecules enter the cells along with the intracellular transfer of inorganic salt ions such as Na^+^ and Cl^−^, resulting in excessive uplift of osmotic pressure, thus forming the pathophysiological process of cytotoxic oedema [50]. This type of oedema does not increase the spacing around blood vessels, but it makes the pressure change significantly in regional microcirculation, which leads to an excessive pressure difference between the precapillary arteriole and postcapillary venule [49]. Then it progresses to the pathological stage of ionic oedema, including the transportation of Na^+^ across the BBB, the formation of the electrical gradient of Cl^−^ and the elevation of the water osmotic gradient. Ionic oedema is characterized by an increase in the net flow of Na^+^ to brain cells [51]. When the damaging effects of the BBB accumulate, permeable pores in the capillary endothelium are formed, and plasma proteins leak into the extracellular space, resulting in vasogenic oedema that can cause significant tissue swelling [49]. Haemorrhagic conversion is the third phase, during which the function of capillary wall collapses, and all of the blood components enter the brain parenchyma, thus leading to a serious haemorrhagic infarction [49]. In fact, haemorrhagic conversion is also considered to be one of the major causes of early death in patients with acute stroke [52].

Additionally, recent studies have proven that AQP4, which is expressed mainly in astrocytes, is a vital channel protein in the progression of brain oedema after TBI [53]. The function of AQP4 is to mediate the flow of water through the BBB and blood spinal cord barrier (BSCB). In a recent study, Kitchen et al. [54] found that the cell-surface abundance of AQP4 increased in response to hypoxia-induced cell swelling in a calmodulin-dependent manner, and CNS oedema was associated with an increase in both total AQP4 expression and subcellular translocation of AQP4 to the BSCB. In other animal studies of TBI and spinal cord injury, researchers found that the calmodulin-inhibiting agent (trifluoperazine) inhibited the localization of AQP4 on the BSCB, thereby promoting resolution of CNS oedema and maintaining the function of the CNS. Meanwhile, Sylvain et al. [55] showed that trifluoperazine inhibited AQP4 expression at both the gene and protein levels, thus effectively reducing cerebral oedema in poststroke mouse models. This study also suggests that trifluoperazine may provide a beneficial extra-osmotic effect on brain energy metabolism via an increase in glycogen levels [55]. This shows that targeting AQP4-mediated brain oedema may be a potential therapeutic strategy for brain oedema after TBI. Generally speaking, these studies provide sufficient evidence for elucidating the post TBI pathological mechanism, and are of benefit in the promotion of drug development.

To date, no obvious evidence has been found to prove the relationship between the long-term prognosis of TBI and changes in the BBB. Further research is needed in related fields.

#### Immune response and neuroinflammation

Based on the above studies, we can speculate that the mechanism of BBB injury is probably linked to perivascular inflammation or autoimmune processes. In fact, some inspiring findings have been revealed previously. TBI activates endothelial cells and elicits dysfunction in mitochondria and glial cells in the regional microenvironment [40–42]. These are known as primary injuries. Activation of endothelial cells then leads to neuroinflammation and an immune response represented by upregulation of cytokine and chemokine levels and recruitment of neutrophils [56]. The activation of innate immune cells, mainly microglia, leads to albumin extravasation and increased BBB permeability [57]. Astrocytes are then activated to secrete matrix metalloproteinases [58], which activate downstream pathways that enhance BBB permeability and contribute to vasogenic oedema after TBI [59]. Monocytes can also activate glial and endothelial cells through the effect of vascular endothelial growth factors [60]. Mitochondrial dysfunction leads to oxidative stress, during which glial cells and neurons release reactive oxygen species (ROS) [61]. ROS can further promote the release of cytokines and chemokines and affect the downstream lipid peroxidation pathway, thereby enhancing paracellular permeability [62]. In particular, a human study suggests that malondialdehyde, a byproduct of lipid peroxidation and oxidative stress, increases immediately after TBI [63]. Additionally, active molecules produced by microglia promote peripherally derived leukocyte adhesion and migration through the endothelial cells of the brain, thus worsening the neuroinflammatory responses in the brain [64]. The reactions mentioned above eventually result in secondary damage to the BBB, which is represented by the interruption of continuity and exudation of blood components.

In addition to the immune response, the effect of TBI on the CNS can also be explained from the perspective of neuroinflammation. Generally speaking, TBI can cause a series of primary and secondary changes in the CNS. The former is represented by damage to the microvasculature and cell membrane, while the latter includes ionic imbalance, calcium overload and mitochondrial dysfunction. These changes lead to mitochondrial stress reactions, excitability toxicity mechanisms and impairment of blood vessels, all of which are assigned to neuroinflammation [65]. Then, cytokines and chemokines are released, and lead to the activation of astrocytes and microglia and the recruitment of circulating immune cells, such as neutrophils, macrophages, and lymphocytes. Activated microglia maintain the activation of other glial cells and neurons in the surrounding microenvironment [66]. After acute TBI, the process above overlapped spatially and temporally [67]. However, the effect of neuroinflammation is two-sided, which means that it plays a role in promoting the process of injury repair, while it can also lead to secondary damage by an excessive inflammatory response. This is determined by the functional plasticity of inflammatory cells in the nervous system. Microglia, for example, are activated to M1 phenotype that produces proinflammatory cytokines and chemokines in response to IFN-γ stimulation, or M2 phenotype that produces anti-inflammatory cytokines with enhanced phagocytic activity in response to IL-4 and IL-13 stimulation [68]. To date, some progress has been made in the long-term effects of neuroinflammation mechanisms in TBI. Neuroinflammation can promote the formation of new synapses after trauma [69], and cytokines produced by inflammatory cells contribute to neurogenesis and angiogenesis, which has a neuroprotective effect [70]. Other studies show that severe TBI may lead to oxidative stress, which induces chronic persistent neuroinflammation and the long-term activation of microglia, as indicated by the maintenance of high serum levels of a series of cytokines and chemokines [71, 72]. Persistent neuroinflammation can also cause the destruction of white matter, which is associated with subsequent cognitive impairments. Similar mechanisms of neuroinflammation are also involved in the development of neurodegenerative diseases, such as CTE and dementia [71].

**Fig. 2:**
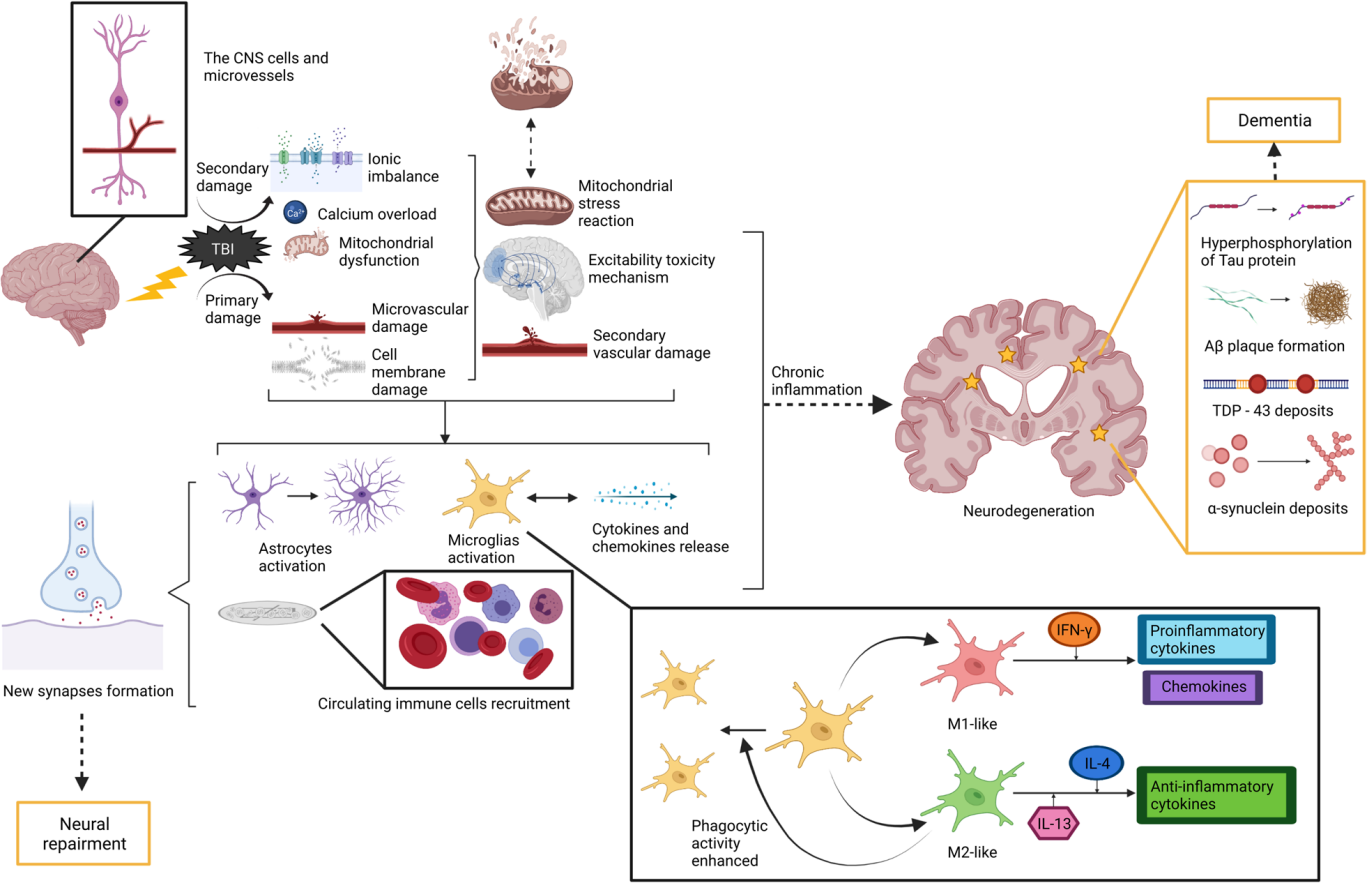
Neuroinflammatory process after the occurrence of TBI and its long-term consequences. After TBI occurs, it can lead to a range of primary (e.g., damage to blood vessels and cell membranes) or secondary (e.g., ion imbalance, calcium overload, and mitochondrial dysfunction) injuries. These injuries together lead to mitochondrial stress cytotoxicity and secondary damage to the vascular system. Subsequently, astrocytes and microglia are activated, and immune cells in the blood vessels are recruited. Microglia can differentiate into M1 and M2 phenotypes, which can produce pro-inflammatory or anti-inflammatory cytokines in response to cytokines such as interferon-γ (IFN-γ), interleukin-4 (IL-4) and IL-13. Microglia itself also divide and play a role in phagocytosis. These neuroinflammatory mechanisms can promote the formation of new synapses, which is conducive to the self-repair of the nervous system. Long-term chronic inflammation can also lead to neurodegeneration, resulting in a series of irreversible pathological changes (such as Tau protein hyperphosphorylation, Aβ plaque formation, TDP-43 and α -synuclein deposition, etc.). Over the years, neurodegeneration can eventually lead to dementia. Solid black and red arrows indicate associations of pathological mechanisms or clinical manifestations, dashed black arrows indicate relationships among subtypes, and dashed red arrows indicate possible aetiology of CTE. TBI traumatic brain injury, CNS central nervous system, Aβ amyloid-β, TDP-43 trans-reaction DNA-binding protein 43 kD

It is worth noting that samples of the human brain can only be obtained from cadavers or surgeries. Therefore, most of the above studies are preclinical, with only one human study mentioned. The role of the immune response and neuroinflammation after TBI and its associations with the neurodegenerative process are shown in Fig. 2.

#### Other pathological mechanisms

Previous studies have shown that TBI can lead to various of neuropathologic changes, including the formation of amyloid-β (Aβ) plaques and trans-reaction DNA-binding protein 43 kDa (TDP-43), α-synuclein accumulation, NFTs, and hyperphosphorylation of Tau protein [73]. Actually, the hyperphosphorylation of Tau protein and NFTs in the brain contributes to concussion symptoms [74], the severity of which is paralleled with the degree of axonal injury.

Tau protein is a microtubule-associated protein with 80 serine/threonine and 5 tyrosine phosphorylation sites. Hyperphosphorylation of Tau protein leads to a threefold increase in its stoichiometry and aggregation into pairs of helical filaments, eventually forming NFTs [75]. Tau hyperphosphorylation has been found in the brain tissue of TBI patients [27]. It should be noted that Tau hyperphosphorylation is also a common neuropathological change in other neurodegenerative diseases, such as AD [76], dementia with Lewy bodies [77], frontotemporal dementia [78], and progressive supranuclear palsy [79]. Although the experimental evidence is still insufficient, we can speculate that TBI and the above types of dementia may be intrinsically linked through the hyperphosphorylation of Tau protein. The mechanism is reflected in a self-perpetuating and progressive neurodegenerative cascade in typical individuals [38]. Thus, it is conceivable that therapies for dementia might also play a role in treating post TBI symptoms, which may become an attractive field in military TBI.

Aβ plaques are commonly associated with AD [80], while α-synuclein is relevant to Parkinson's disease [81]. Researchers also found evidence of TDP-43 in patients with amyotrophic lateral sclerosis and frontotemporal dementia [82]. These findings generally suggest that the long-term adverse neurological outcomes of chronic TBI are associated with an increased risk of dementia. However, the specific role of these pathological substances in TBI and dementia may be different. Aβ is a pathological substance that is thought to be a product of axonal trauma. Aβ plaques in brain tissue and Aβ molecules in cerebrospinal fluid (CSF) may appear within 2–12 h after TBI and are widely distributed [83]. In patients with AD, however, Aβ plaques are more densely packed, and Aβ molecules are rarely detectable in CSF samples. This suggests that Aβ plaques form rapidly in TBI patients, while in AD patients, Aβ molecules take a longer period of time to accumulate. In addition, Aβ plaques can be found in the brain tissues of most AD patients, while the deposition of Aβ occurs only in a relatively small number of patients with TBI [84]. The reason for this may be related to genetic polymorphisms of endogenous enkephalin enzyme [85] and apolipoprotein E [86]. Moreover, in both TBI and AD cases, the dominant type of Aβ in the brain tissue is Aβ42 [83]. Previous studies have confirmed that mutations of the PS1 gene, which is closely related to the occurrence of AD, can lead to the deposition of Aβ42 in brain tissue [87]. Therefore, this kind of Aβ draws special attention. In addition, more fibrous and thioflavin-S positive plaques were found in patients with chronic TBI [84], which may be attributed to the self-repair process of the nervous system after TBI. However, more evidence in relevant fields is still needed.

TBI disturbs glymphatic clearance of brain metabolic waste products, which has been demonstrated in recent studies [88, 89]. This clearance is of particular importance since there is a strong association between TBI-induced glymphatic impairment and the increasing incidence of neurodegenerative diseases among the elderly and military personnel. When entering the brain, CSF goes through the glymphatic system [90]. The system, which is a brain-wide network of perivascular pathways, plays a role in clearing interstitial solutes of the brain [91]. Through animal experiments, it was determined that glymphatic functions are closely related to AQP4. Defects in the AQP4 gene slowed CSF tracer influx and interstitial tracer efflux in mice [92], and hindered the clearance of Aβ [92], thereby promoting Aβ plaque formation [93]. In a more recent study, Mestre et al. [94] found that perivascular AQP4 played a key role in AQP4-dependent glymphatic exchange. These studies provide a basis for further elucidating the mechanisms of chronic TBI and other neurodegenerative diseases, as well as the development of targeted drugs.

It should be noted that the boundary between chronic TBI and CTE is obscure, and the evidence to prove the differences in pathological changes between them is inadequate. Thus, we treated these two concepts equally in the review. In addition, most of the TBI cases involved in these neuropathological studies belong to nonmilitary personnel, so possible differences between military and nonmilitary personnel cannot be ruled out. A summarized illustration of the pathological similarities and differences between TBI and other neurodegenerative diseases is shown in Table 1.

**Table 1:** Pathological similarities and differences between TBI and other neurodegenerative diseases

Diseases	Abnormal p-Tau	Aβ Plaque formation	TDP-43 deposits	α-synuclein deposits
TBI/CTE	**√**	**√**	**√**	**√**
AD	**√**	**√**	**√**	
FTD/FTLD			**√**	
Others*			**√**	**√**

In the above studies, the subjects were not limited to military personnel, so the possibility of negative results in the population of military TBI patients cannot be ruled out

*TBI* traumatic brain injury, *CTE* chronic traumatic encephalopathy, *AD* Alzheimer's disease, *FTD* frontotemporal dementia, *FTLD* frontotemporal lobe degeneration, *p-Tau* phosphorylated Tau protein, *Aβ* amyloid-β, *TDP-43* trans-reaction DNA-binding protein 43 kD

^*^Includes other neurodegenerative diseases such as Parkinson's disease and amyotrophic lateral sclerosis

## Diagnostic technologies

Early diagnosis is important for military TBI. On the one hand, existing research has confirmed the pathological and molecular basis of TBI, which provides potential therapeutic targets. On the other hand, military TBI may lead to a series of unoptimistic consequences, including mood disorders, cognitive impairment, hypoattentiveness and even suicide [21]. TBI may also reduce the quality of life among veterans and elicit a serious impact on their social functions. The concealment of mild TBI, the unpredictability of pathological changes and the delay in a postinjury assessment are the main factors that limit the early diagnosis of military TBI [95]. Eyewitness statements and casualty self-reports are the only available tools to diagnose military TBI in critical situations when no professionals are present on site. However, it is unfortunate that clinicians often lose access to these two aspects of information in a short period of time, especially in the battle environment.

Clinical scales and neuroimaging techniques are expected to provide valuable evidence for the diagnosis of military TBI (Table 2). Although the neuropsychiatric symptoms are relatively complex, most soldiers with acute TBI have transient or persistent brain dysfunction, so the GCS can be used to initially determine the severity of TBI. However, the scale still has some limitations. It can only be used as a cross-sectional judgement of the state of the soldiers in a short time after TBI [96]. Under these circumstances, to better predict the prognosis of patients, scholars have proposed that it is necessary to know the highest GCS score obtained within 24 h after TBI [97].

**Table 2:** Neuroimaging findings among TBI patients with the help of different techniques

Neuroimaging technologies	Discoveries
CT & MRI	The complex multivariate model of CT parameters is helpful to improve the accuracy of prognosis prediction [98]
	The thalamic nucleus volume increased in TBI veterans with suicidal tendencies. The same results were not seen in TBI veterans without suicidal tendencies [99]
	SWI is more suitable for detecting subtle lesions or pathological abnormalities, and is more likely to obtain positive findings [100]
fMRI	The DMN is changed in patients with military mTBI. Military victims with mTBI had more functional connections between white matter and the anterior cingulate cortex [101]
	The posterior cingulate cortex and other posterior brain structures tend to show lower functional connectivity within the DMN [102]
	Patients with mTBI had significantly reduced activity in the right dorsolateral prefrontal cortex (decreased blood oxygenation level dependent effects in the resting state) [103]
	Patients with a reported history of mTBI showed higher activation in the periaqueductal grey matter, right dorsolateral prefrontal cortex and cuneus during pain anticipation [104]
DTI	The radial and axial diffusion coefficients of white matter were significantly increased in TBI group [105]
	Anisotropic dispersive clusters were found in the inferior frontal white matter of mTBI patients [106]
	The prognosis of patients with severe craniocerebral injury was related to the ADC values of the whole white matter and corpus callosum [12]
MTI	The MPF based on magnetization transfer effect decreased in the corticocortical subcortical tracts of patients with explosion-related TBI, and the degree of reduction was correlated with the degree of explosion exposure [37]
ASL	Cerebral blood flow decreased in severe TBI and increased in patients with acute mTBI [107]
MRS	Decreased metabolism of N-acetyl aspartate after concussion [108]
	In veterans, n-acetyl aspartate/creatine and N-acetyl aspartate/choline ratios were reduced [109]

In the above studies, the subjects were not limited to military personnel, so the possibility of negative results in the population of military TBI patients cannot be ruled out

*TBI* traumatic brain injury, *mTBI* Mild traumatic brain injury, *CT* computed tomography, *MRI* magnetic resonance imaging, *fMRI* functional magnetic resonance imaging, *SWI* susceptibility weighted imaging, *DTI* diffusion tensor imaging, *MTI* magnetization transfer imaging, *ASL* arterial spin labelling, *MRS* magnetic resonance spectroscopy, *ADC* apparent diffusion coefficient, *DMN* default mode network, *MPF* macromolecule proton fraction

Acute military TBI is characterized by multifocal TAI and brain oedema. These pathological changes are anatomically manifested mainly as macroscopic cerebral and subarachnoid haemorrhage, while some patients may present with cerebral infarction [38], which can be diagnosed by traditional imaging techniques such as computed tomography (CT) and magnetic resonance imaging (MRI). However, the lack of specificity and difficulty of localization during the acute phase limits the application of these technologies. More importantly, traditional imaging techniques are insufficient for the diagnosis of subtle lesions of brain structure, which urgently needs to be solved. Recently, with the development of functional neuroimaging technology, pathological evidence that has failed to be observed previously can be found in many neuropsychiatric diseases. Functional neuroimaging may become an important adjunctive technology for the localization diagnosis of mental disorders and promotion of precision therapeutic intervention. Currently, diffusion tensor imaging (DTI) and functional magnetic resonance imaging (fMRI) have been employed to diagnose military TBI. However, there are still unavoidable limitations of neuroimaging techniques, such as undetectable subtle changes in neural pathways [110], isotropic results that do not support hypotheses [111], and the most important, more time and cost burden for patients.

Biomarkers can provide a basis for the diagnosis of military TBI and the grading of severity, as well as information on possible targets for drug development. In addition to the changes in glucose metabolism in brain cells, the levels of plasma tyrosine kinase, S100-B, A-II and other biomarkers are conducive to the early diagnosis of military TBI. Meanwhile, some new biomarkers can be applied in the prediction, diagnosis, clinical grading and prognosis judgement of military TBI. Table 3 summarizes the emerging biomarkers associated with TBI. These biomarkers can indicate the responses of brain tissue after TBI, such as haemorrhage, cerebral oedema, focal infarction and skull fracture [112]. In paediatric TBI cases, Berger et al. [113] found that serum neuron-specific enolization enzyme (NSE) and myelin basic protein (MBP) were obviously increased in the TBI group compared with those without TBI. These two biomarkers might be used as forward-looking indicators to judge the existence of TBI and assess the extent of TBI in the future. Computerized neurocognitive assessment tools have been applied to assess the degree of cognitive dysfunction in soldiers with mTBI, but the reliability and validity of this instrument have not yet been verified [20].

**Table 3:** Emerging biomarkers of TBI

Mechanism	Biomarkers	Sample source	Detection technology	Utility^*^	References
Damage of neuron cell body	UCH-L1	Serum	Sandwich ELISA	1	[114]
	NSE	CSF	Electro chemiluminescent assay		[115]
Damage of astrocytes	GFAP	Serum	Sandwich ELISA	1, 2, 3, 4	[116]
	S100-B	Serum; CSF	Automatic electrochemiluminescence immunoassay	1, 2, 4	[117, 118]
Death of neuron	SBDPs	CSF	Bicinchoninic acid microprotein assays	1	[119]
Damage of axon	NF proteins	CSF	ELISA and size-exclusion chromatography and mass spectrometry	1, 2, 4	[120]
Damage of white matter	MBP	Serum; CSF	ELISA	1, 2	[121]
Post-injury neurodegeneration and autoimmune response	Total Tau and phospho-Tau	Brain tissue	Immunohistochemical analysis	1, 2, 4	[122]
	Brain antigen-targeting autoantibodies	Serum	ELISA	3, 4	[123]
Chronic neuronal dendrite regeneration	MAP-2	Serum	ELISA	4	[124]
Survival and regeneration of neurons and axons	BNDF	Serum	Electro chemiluminescent sandwich immunoassay	4	[125]
	NRGN				[126]
	VEGF, etc.		Automatic clotting instrument, etc		[124]
Damage of brain	miRNA	Serum	TaqMan microRNA assays and RT-PCR	1, 5	[127]
	HIF-1α		Immunofluorescence staining		[118]
	Caspase 3 and C5b-9				[118]
Damage of neuron^**^	CNA	Serum	PCR	1, 2	[128]
	Exosome and microvesicles	Serum; CSF	Precipitation reagent and nano sight imaging technology		[129]

In the above studies, the subjects were not limited to military personnel, so the possibility of negative results in the population of military TBI patients cannot be ruled out

*UCH-L1* Ubiquitin carboxy-terminal hydrolase L1, *NSE* Neuron specific enolase, *GFAP* Glial fibrillary acidic protein *S100-B* Central nervous system specific protein-B, *SBDPs* αII-spectrin breakdown products, *NF Proteins* Neurofilament proteins, *MBP* Myelin basic protein, *MAP-2* Microtubule-associated protein-2, *BNDF* Brain derived nerve growth factor, *NRGN* Postsynaptic protein neurogranin, *CNA* Circulating nucleic acids, *PCR* Polymerase chain reaction, *RT-PCR* Reverse transcription-polymerase chain reaction, *VEGF* Vascular endothelial growth factor, *HIF-1α* Hypoxia-inducible factor-1 alpha, *C5b-9* Terminal complement complex, *CSF* Cerebrospinal fluid, *ELISA* Enzyme-linked immunosorbent assay, *Ref* References

^*^The utility here refers only to potential utility, and its accuracy is questionable because most biomarker studies are only experimental and have not been applied in clinical settings. Diagnosis = 1; Prediction = 2; Clinical Classification = 3; Chronic Monitoring = 4; Death Review = 5

^**^Total free DNA levels in patients' plasma are an independent predictor of traumatic death in cases of severe traumatic brain injury

## Tentative treatments

At present, a considerable number of studies on treatments of TBI have been carried out. Compared with symptomatic treatment of neuropsychiatric symptoms, it is more urgent to develop therapies for aetiological treatment. However, TBI, especially mild TBI, is usually subtle, extensive, and difficult to locate directly. Treatments directly targeting intracranial lesions have not been developed yet. Currently, the main interventional approach for military TBI is still aimed at managing neuropsychiatric symptoms rather than promoting full recovery.

A systematic review on the treatment of TBI showed that clinical drug therapies mainly targeted the neurotransmitter system, such as dopaminergic, serotonergic, acetylcholinergic, and glutaminergic neurotransmitter systems [130]. These drugs can work because existing studies have shown that dysfunction of neurotransmitter pathways contributes to the onset of post TBI symptoms [131]. Although there has been some progress in treating the neuropsychiatric symptoms of TBI with nutritional supplements, nootropic drugs, and herbal medicines, such as acetyl carnitine [132], ginkgo [133], citicoline [134], racetam derivatives [135], and omega-3 fatty acids [136], the evidence is still insufficient. At present, drugs that have been entered into clinical studies include methylphenidate [137], amphetamine and cholinesterase inhibitors [138], and a few preliminary results suggest that these drugs have a potentially positive effect on restoring neurological function and promoting rehabilitation.

Pharmacological and physical therapies for TBI remain a research focus. A 2020 study suggested that quetiapine augmentation of prolonged exposure therapy may be beneficial to veterans suffering from TBI and PTSD, but the effectiveness of the treatment has yet to be proven [139]. In a meta-analysis involving 7 randomized controlled trials testing the TBI-treating effect of erythropoietin (EPO), no statistically significant results were found in neurological function improvement and acute hospitalization rate reduction after 6 months; however, the EPO intervention group had a more significant survival benefit than the saline placebo group [140]. A meta-analysis of 6 studies found that although the experimental group showed significant improvement in the GCS score, the treatment efficacy of magnesium sulfate for severe TBI remained questionable [141]. Other preclinical studies have also made some achievements. For example, based on the detection of biomarkers and the mechanism of Tau phosphorylation, Kondo et al. [142] tried to block monoacylglycerol lipase (MAGL) and *cis*-phosphorylated Tau protein with the help of mouse monoclonal antibodies to reduce the neuropathological changes of TBI in 2015, which eventually obtained positive results. The study also sheds light on the potential link between neural stress-induced *cis*-phosphorylated Tau protein and TBI, CTE and AD. However, there are still considerable steps forwards in drug development. Regardless, it should be noted that most of the studies fail to distinguish military personnel as an independent group of TBI patients, and effective therapies for TBI in civilians may not be equally applicable to military personnel.

Transcranial magnetic stimulation (TMS) is the main physical therapy that has been used in clinical practice for neuropsychiatric diseases. Although it may work, the effects of TMS remain to be explored [130]. As a new physiotherapy, transcranial near-infrared light therapy (NILT) can be used to treat skin ulcers, osteoarthritis, myocardial infarction, peripheral nerve injury and other diseases [143] and can even be used to induce stem cell generation [144]. Currently, one study on NILT has displayed a surprising effect in reducing TBI-related symptoms [130]. In a clinical trial, Harch et al. [145] used 100% pure oxygen at 1.5 times atmospheric pressure as "hyperbaric oxygen therapy" in the treatment of 16 soldiers with TBI. They found that the treatment improved a range of post TBI neuropsychiatric symptoms, such as sleep disturbance, restlessness, and headache, as well as the cognitive level of the patients. This improvement suggests that hyperbaric oxygen therapy may facilitate recovery from post TBI symptoms, promote neurocognitive function, and assist in treating comorbidities. Recently, similar statistically positive findings were found in a study conducted by Harch et al. [146]. However, the number of studies in this area is still inadequate, and the available evidence is insufficient to include hyperbaric oxygen therapy as a conventional treatment for military TBI.

Rehabilitation treatments can improve the neuropsychiatric symptoms of soldiers with TBI and restore part of their social functions [95]. Cognitive rehabilitation and behaviour remedy therapy are commonly-used rehabilitation treatments. The effect of these two is to improve cognitive and social impairment [147] that often occurs in TBI patients. It is inspiring that these treatments sometimes show satisfactory efficacy. In addition, through research on nerve neuroregeneration and plasticity, it is expected that functional recovery can be achieved in military personnel with TBI [148]. Despite this, serious consequences of TBI still cannot be ignored. Neurological sequelae, mostly resulting from untimely treatment, include cognitive impairment, multiple pain and seizures [101]. Rehabilitation treatments include neurocognitive rehabilitation and community comprehensive rehabilitation, with the ultimate goal of reintegrating community function and occupational employment for soldiers with TBI [137]. A study by Vanderploeg et al. [149] on the rehabilitation of veterans with acute TBI suggested that patients who received short-term cognitive instructional therapy achieved more significant improvement in neurocognitive function than those who received functional experiential therapy. The benefit of rehabilitation on improving outcomes for TBI soldiers facilitates the transition from military to civilian smoother and paves the way for future policy reforms in military health systems across different countries.

Since 2005, the Polytrauma System of Care has been funded by the U.S. Congress to provide brain injury rehabilitation services to service members or veterans. This system includes the Multiple Trauma Rehabilitation Centre, the Multiple Trauma Website, the Multiple Trauma Support Clinic Team, and the Multiple Trauma Contact Station [150], provides military TBI rehabilitation expertise and draws on the expertise of the Department of Veterans Affairs TBI Leadership Centre, whose history of providing brain injury rehabilitation among veterans dates back to 1991. Additionally, to make veterans with TBI return to normal life, the Polytrauma System of Care also provides one-on-one case management, care services and a special network for rehabilitated veterans to contact their fellow servicemen who are still in service, as well as providing housing for their family members [137].

Medical services should aim to reduce the sequelae of TBI, since the purpose of TBI rehabilitation is to restore the social functioning of soldiers. The Neurobehavioral Symptom Questionnaire, developed by Cicerone et al. [151] in 1995, contains 22 questions to assess the severity of TBI sequelae, and can be used to assist in guiding nursing care. This scale, funded by the U.S. Veterans’ Health System since 2007, has been widely used in a TBI screening program for veterans (mainly those enrolled in OEF and OIF) [137]. To provide further medical services, the U.S. The Veteran Health Administration (VHA) has also funded quality promotion plans, multiple trauma quality improvement research plans and explosive damage research plans, aiming at constructing a comprehensive rehabilitation treatment system.

## Comorbidity and sequelae: another challenge

Neuropsychiatric sequelae such as cognitive impairment, depression, anxiety, neuroendocrine disorders and sleep disorders, may occur just a few months after TBI. The occurrence of sequelae shows no correlations with certain types of military TBI. Even when the injury had been supposed to be clinically cured, these sequelae can still exist, which can seriously affect the physical and mental well-being of the soldiers [152]. This result may be attributed to perturbed neurotransmitter signalling pathways [15] and the deposition of neurotoxic proteins [153]. Although some progress has been made, the specific mechanism needs to be studied further. It should be emphasized that "comorbidities" and "sequelae" are two different concepts. The former focuses on other neuropsychiatric disorders that are associated with TBI, and can be diagnosed solely by existing criteria, but it is not closely relevant to TBI. The latter focuses on a series of neuropsychiatric symptoms following TBI and the causal relationship with the brain trauma itself. However, most studies have not made a clear distinction between the two, so comorbidities and sequelae will be discussed together as follows.

Epidemiological studies show intuitive figures. A meta-analysis showed that the incidence rate of depression and bipolar disorder following mTBI ranged from 10 to 77% [95]. Among U.S. soldiers with mTBI, the average probability of being diagnosed with depression 4 years after TBI was as high as 34%, and the average diagnosis rate of PTSD was as high as 47.8%. The congruent diagnosis rate of depression and PTSD was 73.4%, and the diagnosis rate of PTSD without depression was 12.5% [154]. A systematic review showed that the incidence of PTSD after TBI was significantly higher in the military than in civilians [155], suggesting that there was a correlation between TBI and PTSD due to the military environment. In a survey of 2525 U.S. Army infantry soldiers who were deployed to Iraq for 1 year, Hoge et al. [156] concluded that 44% of soldiers with a history of mTBI met the diagnostic criteria for PTSD.

There may be a neuropathological link between dementia and TBI. A recent review showed that moderate to severe TBI increases the risk of AD, which may be due to blast and chemical exposure [157]. In addition to phosphorylated Tau protein and NFTs, studies have confirmed evidence of axonal damage in the brains of AD and TBI patients [158], namely, increased deposition of toxic proteins [159]. In a retrospective cohort study of 188,764 U.S. military veterans, Barnes et al. [160] found that 16% of those with a history of TBI were diagnosed with dementia in the following 9 years, compared with only 10% of those who never experienced TBI. Furthermore, prospective and retrospective studies have shown that patients with a history of moderate and severe TBI are more likely to develop dementia than those without it, while among patients with dementia, a significant proportion had a history of moderate and severe TBI [161]. In a study of brain injury secondary to explosive exposure among nonhuman primates, chromatolysis in medial temporal lobe hippocampal pyramidal neurons was found by Lu et al. [35]. Since these neurons are responsible for memory processing, dementia following TBI may be associated with degeneration of hippocampal neurons.

Other studies have confirmed that individuals with a history of substance abuse have an increased risk of recurrent substance abuse or dependence after TBI, but there is still a lack of evidence to prove TBI as a risk factor for substance abuse [162]. Schizophrenia is a severe mental disorder, whose classical neuropathological characteristics includes the impairment of the dopaminergic neurotransmitter pathway in the prefrontal cortex and subcortical limbic system. A cohort study of 3495 TBI patients in 2001–2002 found a 1.99-fold increased risk of schizophrenia within 5 years after the occurrence of TBI [163], suggesting that TBI might somehow be a risk factor for schizophrenia. However. the universality of the findings in military personnel remains to be confirmed.

## Discussion

TBI is essentially an organic disease with disruption in neurotransmitter pathways and nerve fibres. TBI is a kind of neurological disease, although it also manifests with a series of psychiatric symptoms. To some extent, it reminds us that a biological foundation might exist for mental disorders. Subtle lesions of local neural structures may be the root cause of some mental disorders, so they cannot be defined as "functional". However, we still cannot deny the contributing role of other susceptibility factors on the pathogenesis of mental disorders, such as heredity, environment, unhealthy lifestyle, and other physical diseases or neurological degenerative changes with unclear aetiology. Perhaps in the near future, neurology and psychiatry will no longer be separated from each other.

The establishment of diagnostic criteria is rather difficult [164], especially in the last century. Even today, immediate interventions in patients with acute brain injury are still a clinical challenge despite various new technologies [95]. Although the pathological mechanism changes of TBI have been gradually clarified, clinical therapies remain mainly symptom-driven. To fully improve injuries within the nervous system and enable soldiers to regain their social functions, further technological development of relevant diagnosis and treatment is urgently warranted.

During World War I, the concept of TBI was used to judge whether soldiers could continue their military service; however, now, it is widely recognized as an organic trauma characterized by various neuropsychiatric symptoms. Existing research evidence has primarily elucidated the correlation between TBI and other neurodegenerative diseases [159]. Understanding the mechanism of sequelae of traumatic brain function provides indications for its treatment, but the problem is that the lack of specificity of these mechanisms makes it difficult to develop precise interventions exclusively for TBI.

Acute pathological reactions of neuronal axons and the microvascular system in the brain medulla after exposure to explosive weapons [24] result in NFTs and local Tau phosphorylation in brain tissues [74]. With the assistance of advanced neuroimaging techniques, these pathological processes were characterized by the decreased local metabolic activity of brain tissues [36], changes in functional connectivity between the cerebral medulla and the cingulate cortex [101], and changes in the DTI diffusion coefficient [105]. The hypothesis is that the phosphorylation of Tau protein is the mainstream mechanism that leads to post TBI neuropsychiatric symptoms, but the available evidence is insufficient to correlate Tau phosphorylation and diffuse axonal injury. In recent years, researchers have paid special attention to biomarkers derived from artificial animal models to mimic the pathophysiological changes in TBI [142] and further verified them in patients with a confirmed clinical diagnosis [165]. However, according to the existing research results, there is still a long way to go to clarify these mechanisms.

Therapies for treating TBI also meet challenges. If the injured area cannot be accurately located, attempts to intervene in the sequela of TBI through neurotrophic and psychotropic drugs, or even traditional physical therapies, often turn out to be unsuccessful [140, 141]. Although other newly-developed therapies, such as hyperbaric oxygen therapy and near-infrared laser irradiation have shown positive results [130], these therapies seem to be relatively preliminary and far from clinical transformation. Moreover, another challenge is that those diagnostic techniques and therapies are used mostly for nonmilitary populations, while the differences between military and nonmilitary personnel cannot be ignored. Although TBI and other neurodegenerative diseases cannot be completely cured, the latest research offers new ideas for drug development. Aldewachi et al. [166] pointed out that efficient high-throughput screening may become popular in the process of drug development. This technology can screen a large number of compounds per day to greatly reduce the cost and time. Moreover, in silico libraries, and molecular docking software combined with the upscaling of cell-based platforms have proven the potential to promote screening efficiency with higher predictability and clinical applicability. Furthermore, computer-aided drug design strategies minimize the huge number of ligands that need to be screened in traditional biological assays, thus providing a brand-new method for drug development [167]. Comorbidities are another problem. Several prospective studies have suggested a close association between military TBI and the cooccurrence of other neuropsychiatric diseases [154, 155], although the specific mechanisms have not been well elucidated. Moreover, there is no suitable quantitative parameter to assess the risk of TBI for other neuropsychiatric diseases. Given these challenges, it is critical that the Department of Defence and other scientific communities seek more support for research into TBI and related fields.

Regardless, it is comforting that newly-developed treatments addressing the mechanisms of TBI and other neuropsychiatric diseases are on the way. High-throughput microfluidic devices, for example, can be used to assess the passage of large biopharmaceuticals across the BBB [168]. Salman et al. [169] proposed an in vitro microvascular open model system using human brain microvascular endothelial cells. Compared with other traditional closed microfluidic platforms, this new system has a barrier-like function and can be used to investigate mechanisms of transcytosis across the brain. The system enables real-time monitoring of BBB penetration and permeability during TBI, thus forming a new way to control the post pathological changes of TBI. Based on experiments in mouse stroke models, cerebral organoid transplantation has been proven to reduce brain infarct volume and improve neurological motor function, enhance neurogenesis, synaptic reconstruction, axonal regeneration and angiogenesis, and promote neuronal survival from apoptosis. We can speculate that this method has the potential to treat TBI [170]. In addition, the combination of 3D printing technology and cell engineering also provides new ideas for the treatment of TBI. Chae et al. [171] established a novel therapeutic platform based on 3D cell-printing and tissue-specific bioinks, thus forming a probable solution for functional TBI regeneration. In addition, the establishment of government-funded rehabilitation institutions and standardized organizations within institutions facilitates the management of military TBI. To date, in addition to the military, more civilian TBI patients have received treatment due to timely diagnosis. Thus, the number of patients with severe loss of social function (such as major depression, dementia or neurological insufficiency) due to post TBI comorbidities or sequelae is reduced, as well as the social burden in the long-term future.

## Conclusions

From World War I to the present, military psychiatry has experienced ongoing development. As various military operations continue, instances of TBI in military personnel have gradually received increasing attention from the fields of psychiatry and neurology. However, despite rapid scientific advances, more challenges emerge as well. Immediate intervention, rehabilitation, and the specific intervention of comorbidities and sequelae should be the focus of future research in this field. Assessment of the severity of the trauma and early intervention can improve the quality of life of soldiers with TBI. Promoting the recovery of the social function of soldiers with TBI is a comprehensive and systematic task. Fortunately, new technologies have been developed to improve the prognosis of TBI patients, although some of them are still in the preclinical stage. Overall, prevention is still the most important method. Although the causes are varied, military TBI in essence is an organic neuropathy. Therefore, the intervention of this disease requires the joint efforts of neurology, psychiatry and other clinical disciplines.

## Data Availability

Not applicable.

## Abbreviations

Aβ: Amyloid-β
AD: Alzheimer's disease
AQP4: Aquaporin-4
BBB: Blood brain barrier
BSCB: Blood spinal cord barrier
CNS: Central nervous system
CSF: Cerebrospinal fluid
CTE: Chronic traumatic encephalopathy
DSM: Diagnostic and statistical manual of mental disorders
FTD: Frontotemporal dementia
FTLD: Frontotemporal lobe degeneration
GCS: Glasgow coma scale
mTBI: Mild traumatic brain injury
NFT: Neurofibrillary tangles
OEF: Operation enduring freedom
OIF: Operation Iraqi freedom
OND: Operation new dawn
PCS: Post-concussion symptoms
PTSD: Post-traumatic stress disorder
ROS: Reactive oxygen species
TAI: Traumatic axonal injury
TDP-43: Trans-reaction DNA-binding protein 43 kD
VHA: Veterans health administration
